# Central serous chorioretinopathy with and without steroids: A multicenter survey

**DOI:** 10.1371/journal.pone.0213110

**Published:** 2019-02-28

**Authors:** Takashi Araki, Hiroto Ishikawa, Chiharu Iwahashi, Masanori Niki, Yoshinori Mitamura, Masahiko Sugimoto, Mineo Kondo, Takamasa Kinoshita, Tomo Nishi, Tetsuo Ueda, Aki Kato, Tsutomu Yasukawa, Yoshihiro Takamura, Fumi Gomi

**Affiliations:** 1 Department of Ophthalmology, Hyogo College of Medicine, Hyogo, Japan; 2 JCREST (Japan Clinical Retina Study) group, Kagoshima, Japan; 3 Department of Ophthalmology, Sumitomo Hospital, Osaka, Japan; 4 Department of Ophthalmology, Tokushima University, Tokushima, Japan; 5 Department of Ophthalmology, Mie University Graduate School of Medicine, Mie, Japan; 6 Ophthalmology, Sapporo City General Hospital, Hokkaido, Japan; 7 Ophthalmology, Nara Medical University, Nara, Japan; 8 Department of Ophthalmology and Visual Science, Nagoya City University Graduate School of Medical Sciences, Aichi, Japan; 9 Department of Ophthalmology, Fukui University, Fukui, Japan; International University of Health and Welfare, JAPAN

## Abstract

We investigated the rates of the use of steroids in Japanese central serous chorioretinopathy (CSC) cases and differences in the characteristics of CSC with and without steroids. A total of 538 eyes of 477 patients diagnosed with CSC, with 3 months or more of follow-up between April 2013 and June 2017 at 8 institutions. Patients with CSC with more than 3 months of follow-up were identified by OCT and fluorescein angiography at 8 institutions. Data collected included patient demographics, history of corticosteroid medication and smoking, spherical errors, findings of angiography, subfoveal choroidal thickness, and changes through the follow-up period. Differences in these findings were analyzed in cases with and without corticosteroid treatment. Among the 477 patients (344 men,133 women), 74 (15.5%) (39 men, 35 women) underwent current or prior steroid treatment. Cases with steroids were higher age (p = 0.0403) and showed no male prevalence, more bilateral involvement (p < 0.0001), and the affected eyes had multiple pigment epithelial detachment (p <0.0001), more fluorescein leakage sites (p < 0.0001), greater choroidal thickness (p = 0.0287) and a higher recurrence rate (p = 0.0412). Steroids can cause severer CSC through an effect on choroidal vessels and an impairment of retinal pigment epithelium.

## Introduction

Central serous chorioretinopathy (CSC) is a disease in which serous retinal detachment is seen, often associated with detachment of the retinal pigment epithelium (RPE) [1–10]. Fluorescein angiography (FA) in eyes with CSC reveals one or more leakages through RPE defects, and indocyanine green angiography (ICGA) often shows choroidal vascular hyperpermeability [8,9]. Increases in the permeability of the choroidal vessels result in pigment epithelial detachment (PED) and exudation of fluid into the subretinal space. It typically affects young and middle-aged adults, with men affected more commonly than women. Traditionally, CSC has been found to be associated with psychological stress and type-A personality, but its pathogenesis remains unclear [10].

An association with corticosteroids had been postulated, and since Wakakura and Ishikawa [11] demonstrated 5 cases showing worsening of CSC during corticosteroid therapy in the Japanese-language literature in 1980, more attention has been paid to the risk of steroids with respect to CSC. Not only systemic administration but the local routes of steroids can cause CSC [12–22].

A history of CSC can increase the risk of neovascular age-related macular degeneration (AMD), especially polypoidal choroidal vasculopathy, and recent studies indicate a common feature of thick choroid among these diseases [23–25]. Hence it is desirable to learn more about CSC pathogenesis, including the possible risks of steroids.

In this study, we investigated the rates of the use of steroids in Japanese CSC cases and differences in the characteristics of CSC with and without steroids. In addition, choroidal thickness was compared among patients.

## Participants and methods

This was a multicenter retrospective study (participating facilities: Hyogo College of Medicine, Sumitomo Hospital, Tokushima University, Mie University Graduate School of Medicine, Sapporo City General Hospital, Nara Medical University, Nagoya City University Graduate School of Medical Sciences, and Fukui University). The study was approved by the ethics committees of all participating centers (ethics committee of the Hyogo College of Medicine, number 2387, Sumitomo Hospital, number 29–28, Tokushima University, number 2831, Mie University Graduate School of Medicine, number 3151, Sapporo City General Hospital, number H29-054-364, Nara Medical University, number 1464, Nagoya City University Graduate School of Medical Sciences, number 60160195, Fukui University, number 2017000). The need for written informed consent was waived by the ethics committees due to a retrospective and observational nature of the study. The data acquired in the course of the data analysis were anonymized before we accessed them. The study complied with the Declaration of Helsinki.

In this study, patients who showed submacular fluid associated with CSC in at least one eye and were followed for more than 3 months between April 2013 and September 2017 were included. The diagnosis of CSC was based on ocular examination, a typical fluorescein leakage seen in CSC and subretinal fluid on OCT. Eyes showing the occult type-leakage or irregular, broad elevation of the RPE at the macula, those were indicative of CNV, were excluded. Patients with other retinal and choroidal abnormalities showing intra- and subretinal hemorrhage were also excluded. Medical data on patients’ ages, gender, disease bilaterality, history of steroids, smoking history, and history of medication by antianxiety drug, were collected. Following data were obtained from affected eyes and if both eyes had CSC, we chose one eye with later onset or randomly; best corrected visual acuity, spherical equivalents, the numbers of regions showing leakage on FA, the presence of choroidal vascular hyperpermeability on later-phase ICGA, and subfoveal choroidal thickness were collected as possible. The reading of the angiography was done at individual centers for the purposes of this study using the protocol described. The subfoveal choroidal thickness was obtained from 426 eyes from the collected images of the spectral-domain OCT of Spectralis (Heiderbelg Engineering, Heiderbelg, Germany) or Cirrus (Carl Zeiss Meditec, Inc, Dubrin, CA). This was measured as the vertical distance between the outer border of the RPE and the chorioscleral border with duplicate doctors. In this study, the values of choroidal thickness obtained by means of different instruments were used without adjustment because the error between different instruments has been reported to be less than the inter-observer difference [26].

Major outcomes were the rate of patients who had undergone administration of steroids before they were diagnosed with CSC and differences in characteristics as opposed to eyes without steroid usage.

BCVA was examined using Landort C chart and then converted to logMAR (logarithm of the minimum angle of resolution) for statistical analysis. Analysis was performed with JMP software (Version pro 13, SAS, Cary, NC). To compare the two groups, Student’s t test and Pearson’s chai-square test were performed as appropriate. Multivariate analysis was performed to determine whether the use of steroids significantly affects choroidal thickness. Values are shown as mean±standard deviation. The statistical significance of P values was set at 0.05 or less.

## Results

### Patients’ demographics

We examined the records of 538 eyes from 477 patients (344 men and 133 women, M/F ratio 2.6:1) by CSC in 8 hospitals. The mean age was 53.7 ± 11.3 (range 29–89). Sixty-one patients (12.8%) had bilateral disease. Answers about smoking history was obtained from 345 patients and previous or current smoking was recorded for 147 patients (42.6%). Of those, 135 patients (91.8%) were male. Ten patients took one or another anti-anxiety drug.

### Corticosteroid medication

Seventy-four patients (15.5%; male 39, female 35) had current or prior systemic steroid histories. The number of current users was 49 and of prior users 14, but no information was available for 11 patients. The average periods of steroid use were 51.1 (range 1 to 120) months for the current users and 20.5 (range 1 to 240) months for the prior users.

The major reasons for the use of steroids were systemic lupus erythematosus (12 patients), nephropathy or post-renal transplantation (12 patients), and arthritis (10 patients). A local ointment was prescribed for dermatitis in the case of one male patient. In the case of four patients, systemic steroids were prescribed by an ophthalmologist: for recurrent scleritis in one patient and CSC or chorioretinitis in three patients. Sixteen patients discontinued steroids after the development of CSC.

### Characteristics of affected eyes

The average spherical errors of the affected eyes were -0.61 ± 2.26 diopters (range: -9.0 to + 8.25). Thirty-nine eyes (8.18%) showed multiple PED. At least one region of fluorescein dye leakage within the macula in 235 eyes and multiple fluorescein leakage sites were seen in 225 eyes. ICGA was available for 305 eyes, and 230 eyes (75.4%) showed choroidal vascular hyperpermeability (CVH). Choroidal thickness was measured for 426 eyes, and the average thickness was 375 ± 100 (range, 136–674) microns. Details were shown in S1 Dataset.

### Differences in findings with and without corticosteroid medication

Differences in patient background are shown in Table 1. Cases with steroids showed older age (p = 0.0403), no male prevalence, and more bilateral involvement (p < 0.0001). The number of cases with smoking history was significantly low (p = 0.0345) and that with the history of antianxiety drug was significantly high (p = 0.0016) in the group with steroids.

**Table 1:** Differences in patients backgrounds.

	Total	Steroid (+)	Steroid (-)	P Value
No. of cases	477	74	403	
Age (yrs)				
Mean (SD^a^)	53.7 (11.3)	56.1 (11.8)	53.2 (11.2)	0.0403*
Gender (male/female)	344/133	39/35	305/98	<0.001^†^
Bilateral involvement (%)	61 (12.8)	31 (41.9)	30 (7.4)	<0.0001^†^
Smoking (no. of cases/data available) (%)	147/345(42.6)	13/46 (28.3)	134/299 (44.8)	0.0345^†^
Antianxiety drug (no. of cases/data available) (%)	10/445(2.2)	5/66 (7.6)	5/379(1.3)	0.0016^†^

^a^ SD = standard deviation

* Student’s t test

^†^ Pearson’s chi-square test

A comparison of the characteristics of eyes with and without steroid usage is shown in Table 2.

**Table 2:** Characteristics of eyes with and without steroid use.

	Total	Steroid (+)	Steroid (-)	P Value
No. of eyes (%)	477 (100)	74 (15.5)	403 (84.5)	
Spherical equivalent (diopters)				
Mean (SD^a^)	-0.61 (2.26)	-0.30 (1.70)	-0.67 (2.34)	0.1920*
Best corrected visual acuity (logMAR^b^)				
Mean (SD^a^)	0.12 (0.24)	0.07 (0.25)	0.03 (0.19)	0.1950*
Multiple PED^c^ (%)	39 (8.18)	25 (33.8)	14 (3.5)	< 0.0001^†^
Fluorescein angiography findings				
Data available (n) (%)	464	70 (15.1)	394 (84.9)	
leakage involving the fovea (n) (%)	235 (50.7)	40 (57.1)	195 (49.5)	0.2373^†^
Multiple leakage sites (n) (%)	223 (48.3)	50 (71.4)	173 (44.1)	< 0.0001^†^
Indocyanine green angiography findings				
Data available (n) (%)	305	45 (14.8)	260 (85.3)	
Choroidal vascular hyperpermeablitiy (n) (%)	230 (75.4)	38 (84.4)	192 (73.9)	0.1274^†^
Choroidal thickness (μm)				
Data available (n)(%)	426	66 (15.5)	360 (84.5)	
Mean (SD^a^)	375 (100)	399 (108)	370 (98)	0.0287*

^a^ SD = standard deviation

^b^ logMAR = logarithm of the minimum angle of resolution

^c^ PED = pigment epithelial detachment

* Student’s t test

^†^ Pearson’s chi-square test

**Fig 1:**
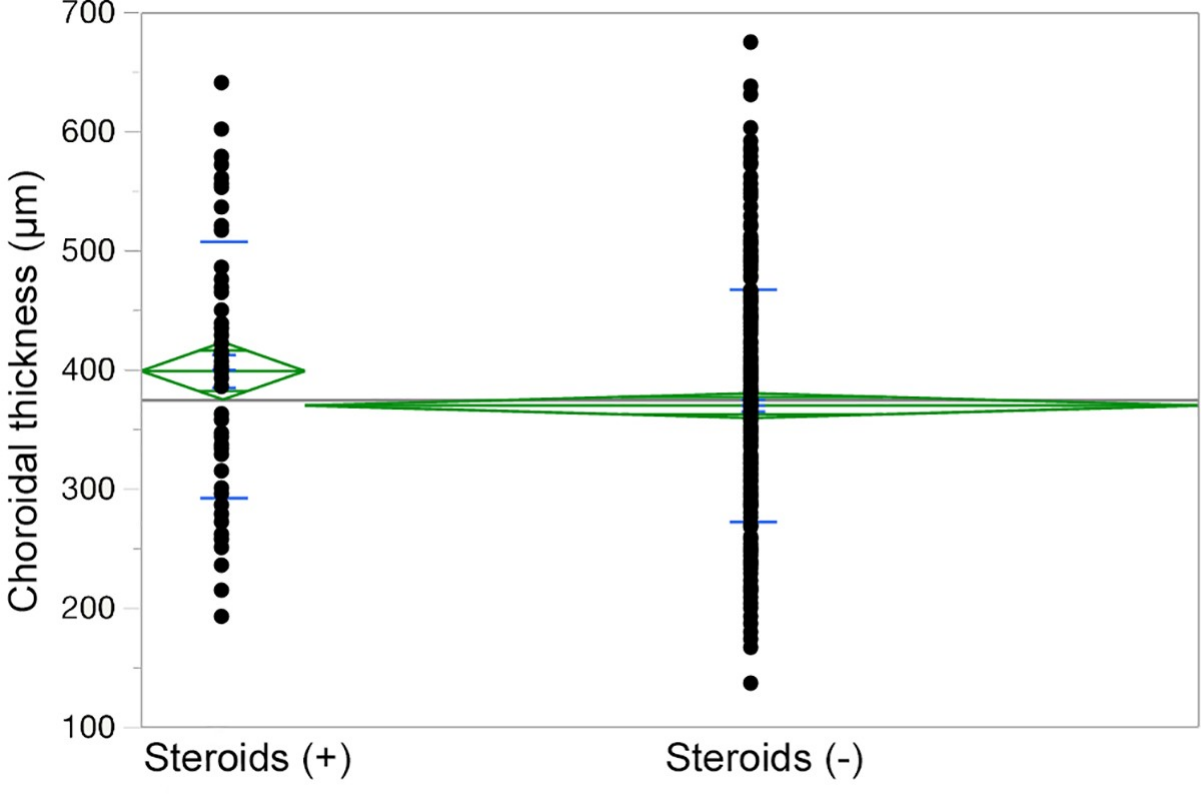
Dot plot of choroidal thickness of eyes with and without steroid use. Legend: Choroid was significantly thick in eyes with steroids.

Choroidal thicknesses were available for 426 eyes from 426 patients, and 66 patients (15.5%) had undergone steroid treatment. Of the 51 eyes for which choroidal thicknesses were not available, 15.7% (8 eyes) received steroid treatment, and thus these two percentages were almost the same. The choroids of the eyes that had received steroid treatment were significantly thicker than those of the patients who had not undergone steroid treatment (p = 0.0287). (Fig 1). The choroidal thickness was not significantly different between the current (391.8 μm) and prior (415.5 μm) steroid users (p = 0.501).

To identify the major factors associated with choroidal thickness, we performed multivariate analysis among the factors of sex, age, steroid treatment, spherical errors, and the presence of choroidal vascular hyperpermeability. The results showed that choroidal thickness was significantly associated with younger age (p < 0.0001), more spherical equivalent (p < 0.0001), the use of steroids (p = 0.0155), and males (p = 0.0482), that is, the steroids independently increased choroidal thickness. No relationship was observed in the case of choroidal vascular hyperpermeability (p = 0.6753).

### Follow-up

The average follow-up period was 14.5 ±14.5 months (range, 3–144 months). During the follow-up period, 366 eyes (76.7%) underwent treatment, including laser photocoagulation in 133 eyes, photodynamic therapy in 220 eyes, and intravitreal anti-VEGF drug injection in 24 eyes. In the cases with steroids, 52 eyes (70.3%) underwent treatment. Laser photocoagulation was done for 29 eyes, photodynamic therapy for 26 eyes, and intravitreal anti-VEGF drug injection for 2 eyes. In the cases without steroids, 314 eyes (77.9%) underwent treatment. Laser photocoagulation was done for 104 eyes, photodynamic therapy for 194 eyes, and intravitreal anti-VEGF drug injection for 22 eyes. Recurrences were recorded in 121 eyes (25.4%), and the rate was significantly higher in eyes with steroids (35.1%) than without steroids (23.6%) (p = 0.0356).

## Discussion

This study included a relatively large number of Japanese patients with CSC, and analysis focused on patient background with and without the use of corticosteroids. Many studies have indicated an association of steroids with CSC. According to a recent systematic review analyzing risk factors for CSC [27], the odds ratio (OR) for steroids was 4.29, and this number was the highest among possible risk factors, including *Helicobacter pylori* infection (OR = 3.12), autoimmune disease (OR = 3.44), psychopharmacologic medication use (OR = 2.69), and type-A behavior (OR = 2.53).

The patients included in this study might have had severer CSC because they were referred by primary clinics and were followed for more than 3 months in one or another of the various hospitals. The average age of the patients was 53.7 years, higher than the 41 [28], 45 [29], and 51 [19] years in previous reports. The male / female ratio was 2.6, and the rate of cases with a smoking history was 42.6%. According to a 2017 survey by Japan Tobacco, Inc. (website of the Ministry of Health, Labor and Welfare in Japan, http://www.health-net.or.jp/tobacco/product/pd090000.html), the average rate of smoking among Japanese was 18.2%, and for males aged 30–50, the rate was about 35%, therefore, CSC patients have higher rate of smoking habit.

The affected eyes were mostly emmetropic, although the myopic population is increasing in Japan. More than half showed multiple leakage points on FA, and more than three-quarters showed choroidal vascular hyperpermability on ICGA. The average choroidal thickness was 375 microns.

Use of corticosteroids was confirmed in 74 cases (15.5%), and the rate was high compared with previous studies, for example, 3.3% [15], 9.1% [28], and 14.4% [29], possibly due to the inclusion of severer cases. More than 60% of the patients were current users, and in 4 cases, steroids were prescribed by ophthalmologists. Bilateral CSC tends to be misdiagnosed as chorioretinitis or Harada disease, as previously reported [20]. The duration of steroid use was relatively long.

In the comparison with and without steroids, several differences were identified. CSC related to glucocorticoids showed lower male predominance, higher age, and more frequent bilaterality at significant levels, as previously reported [22]. The rate of having a smoking history was significantly lower in cases with steroids, while it reached to 44.8% in cases without steroids. Antianxiety drug was taken more frequently in cases with steroids.

On FA, CSC with steroids showed significantly more leakage sites, including the fovea and multiple PEDs. ICGA had a greater tendency to show choroidal vascular hyperpermeablitiy, and the choroid was significantly thicker in eyes with steroid treatment than in those without treatment. The multivariate analysis showed that steroid treatment can increase choroidal thickness independently of age, spherical errors, and gender [30, 31].

Choroidal vascular hyperpermeablitiy and increased choroidal thickness in eyes with CSC indicate the involvement of choroid in pathogenesis [32,33], and our study indicates that steroids can cause CSC via choroidal vascular changes. A previous pilot study evaluating the effect of intravenous drip of steroids on choroidal thickness revealed that high-dose steroids did not alter choroidal thickness, but one of 20 patient developed CSC with increased choroidal thickness [34]. These results suggest that subjects who show susceptibility of steroids can develop CSC with increased choroidal thickness. On the other hand, a recent study comparing choroidal thickness in 25 eyes with CSC secondary to systemic corticosteroid use and with idiopathic CSC showed that the mean central choroidal thickness in secondary CSC was significantly less [35]. They also found a significantly smaller proportion of cases showing choroidal vascular hyperpermeablitiy in secondary CSC, and speculated that corticosteroid might cause CSC by unknown mechanisms that are not related to choroidal thickening. Because our study included a great number of patients treated with steroids, we believe that CSC secondary to steroids can occur through choroidal vascular changes such as congestion of choroidal blood flow. Zhao et al [36]. found that intravitreal injection of the glucocorticoid corticosterone into rat eyes induced choroidal enlargement, and suggested that the underlying mechanism is activation of the mineralcorticoid receptor pathway by glucocorticoids, which induces upregulation of endothelial vasodilatory potassium channel KCa2.3 (calcium-dependent channel) leading to choroid vasodilation.

The prognosis for CSC secondary to steroids is not promising due to the severity of the manifestations. In this study, a larger number of fluorescein leakage sites associated with a thickened choroid indicated diffuse damage of the RPE, which caused more frequent recurrences. Earlier recognition and discontinuation of steroids is necessary to delay visual deterioration.

There were several limitations in this study. Because this was a retrospective study, not all data were available. The onset of CSC was difficult to determine accurately for patients with chronic disease, and hence the association of the duration of steroid use with the development of CSC was not evaluated. Thus the effect of duration of steroid use on choroidal thickness could not be analyzed. Readings of angiography and OCT were not performed at the reading centers and there was no consensus as to treatment.

In spite of these limitations, our study illuminates the role of steroids and CSC via choroidal vascular changes in inducing thick choroid. Further awareness of the risk of CSC due to steroids is to be advised.

## Supporting information

S1 Dataset Original data used in this article are included. (PDF)
