# Supplementary material for: Central serous chorioretinopathy with and without steroids: A multicenter survey
Source: PLoS One. 2019 Feb 28;14(2):e0213110. doi: 10.1371/journal.pone.0213110 (PMC6394983; doi:10.1371/journal.pone.0213110)
Supplement: S1 Dataset — (PDF) [file pone.0213110.s001.pdf]

case	age	gender M=0, F=1	R =0 or L =1	SE	CSC=0, MPPE=1	both eyes	Steroids (+0, -1)	smoking (+0, -1)	antianxiety drug(+0, -1)	location of leakage	number of leakage (1=0, within3=1, morethan4=2)	CVH on IA (+0, -1)	SFCT(microns)	recurrences (+0, -1)	Baseline BCVA
1	40	0	1	-4.0	1	1	0		1				242	0	0.2
2	59	0	0	-1.5	1	1	0		1	1	1		392	1	0.15
3	68	1	1	1.5	0		0		1	1	1	0	214	1	1.2
4	69	1	1	-3.5	0		0		1	0	0	0	235	1	1
5	80	0	0	-2.0	0		0	1	1				286	0	1
6	70	0	1	1.3	0		0	1	1	0	1	0	300	0	0.8
7	63	1	0	-1.0	0		0			1	0	0	428	1	1.2
8	68	1	0	1.5	1	1	0	1		2	1	0	414	0	0.7
9	60	1	0	-0.8	1	1	0			2	1		285	1	1
10	55	0	0	0.0	0		0			1	1		249	0	0.8
11	65	1	0	2.5	1	1	0	1	1	2	1	0	314	0	0.8
12	65	1	0	-2.0	0	1	0		0	1	0	0	278	0	1
13	64	1	1	2.5	1		0	1	1	2	1	0	397	0	1.5
14	54	1	0	-1.5	0		0	1	1	0	1	0	192	0	1.2
15	53	0	0	1.5	1	1	0	1	1	0	1		278	0	0.8
16	64	1	0	-1.0	0	1	0	1	1	1	1	0	428	1	1.2
17	49	1	0	-4.0	1		0		1	1	1		571	1	1
18	63	0	0	0.3	1	1	0	0	1	2	1	1	400	1	0.6
19	73	0	1	-1.8	1		0	1		1	1	1	271	0	1
20	66	1	0	0.3	0		0			1	1		343	0	1.2
21	47	1	1	-2.8	0		0	0	1	1	1	0	464	1	0.4
22	64	0	1	0.3	0		0	0	1	1	1		300	1	1
23	70	0	0	0.8	1	1	0		1	1	1		485	1	1
24	50	1	0	0.0	0		0	1	1	1	1	0	536	0	0.2
25	52	0	0	0.0	0		0	1	1	1	1		286	1	0.5

26	46	0	0	0.5	1		0		1	1	1		271	0	0.2
27	46	1	0	-0.3	0		0	0	1	1	0	0	560	0	0.9
28	69	0	0	-1.5	DRPE	1	0	1	1	2	1	1	362	0	0.4
29	43	0	0	-2.0	1	1	0	1	1	2	1	0	257	1	0.1
30	49	0	0	-1.0	0	1	0		1	1	1	0	421	1	1.2
31	78	0	0	-7.3	DRPE	1	0	0	1	2	1	0	362	0	0.2
32	47	0	0	-0.8	1	1	0	0	1	2	1	0	571	1	0.1
33	34	0	0	-0.5	0		0	1	1	1	1	0	336	1	1.0
34	42	1	0	0.0	1	1	0		1	2	1		428	1	0.7
35	61	1	0	1.5	0		0	1	1	0	1	0	554	1	1
36	44	1	0	0.3	0		0	1	1	1	0	0	357	1	0.7
37	55	1	0	-3.0	0	1	0	1	1	0	1	0	328	1	0.5
38	44	0	0	-7.3	0		1		1	1	1		250	1	1
39	44	0	1	0.5	0		1		1	1	0		571	0	1.2
40	54	0	0	0.5	0		1	0	1				321	1	1
41	39	1	0	-1.8	0		0			1	0	0	250	0	1
42	73	0	9	0.2	0		1	1	1	1	0	0	257	1	0.4
43	53	0	1	-1.6	0	1	1	1	1	0	1	0	371	1	1
44	46	0	1	-1.5	0		1			1	0		186	1	1.2
45	52	0	1	0.0	0		1	0	1	1	0	0	357	1	1.2
46	55	1	0	0.5	0		1	1	1	1	0		336	1	1.2
47	62	0	1	1.0	0		1	1	1	0	1	0	364	1	0.7
48	60	0	1	0.8	0		1		1	1	0		406	1	0.6
49	49	0	1	-0.5	0		1	1	1	0	0	0	250	0	0.8
50	68	1	1	-3.0	0		1			1	1	1	300	0	1
51	54	1	0	0.0	0		1			1	0	0	136	0	1
52	60	0	1	0.8	0		1		1	0	0		357	0	0.7
53	42	0	1	0.0	0		1		1	1	0	0	392	1	0.9
54	38	0	0	-2.0	0		1			1	1		214	1	1.2
55	53	0	1	0.3	0		1		1	0	0	0	357	1	0.6
56	49	0	0	-4.6	0	1	1	0	0	2	1	0	450	1	0.8
57	48	0	0	0.0	0		1			1	1		357	0	0.8
58	45	0	1	-6.0	0		1			1	0		250	0	1.5

59	38	0	0	-0.5	0		1		1	0	0	0	435	0	1.2
60	44	0	1	1.0	0		1		1	1	1		457	1	0.7
61	50	0	0	0.0	0	1	1		1	2	1		300	0	1.2
62	36	1	0	1.0	0		1	0	1	0	1		300	0	1.2
63	69	1	0	-2.4	0		1			1	1	0	300	0	0.7
64	41	0	0	-1.3	0		1			1	0		321	1	0.5
65	53	1	0	-1.8	0		1		1	0	0	0	307	1	0.8
66	39	0	1	0.0	0		1		1	0	0		216	0	0.9
67	39	0	1	-4.6	0		1	0	1	1	0	0	357	1	1.0
68	47	0	0	-3.5	0	1	1		1	1	0		228	1	0.4
69	50	0	0	-0.5	0		1		1	1	1		371	0	1.2
70	81	1	0	1.6	0		1	1	1	1	1	1	321	1	0.6
71	66	0	0	1.8	0		1			1	1	0	300	0	0.5
72	44	0	1	0.5	0		1		1	1	0		449	1	1.2
73	43	0	0	-9.0	0		1		1	1	0		508	1	0.9
74	42	0	0	3.3	0		1		1	1	0	0	585	0	1
75	56	0	1	-0.8	0		1			1	1		250	0	0.9
76	39	0	0	-0.8	DRPE	1	1	0	1	1	0	0	335	0	0.8
77	55	1	1	1.6	0		1		1	1	0		314	0	0.8
78	58	0	0	-0.9	0		1		1	0	0		333	1	0.5
79	59	1	0	2.0	0		1		1	1	0		364	0	1
80	47	0	1	-0.5	0		1		1	1	0		349	0	1.2
81	49	1	1	-7.1	0		1	1	1	1	0	0	192	1	1
82	43	0	1	-5.9	0		1	1	1	1	1	0	392	1	1.2
83	51	0	1	-3.5	0		1		1	1	2	0	328	1	0.3
84	62	0	1	-3.5	0		1		1	1	1	0	221	1	0.15
85	41	0	1	-1.5	0		1		1	0	0		378	1	0.5
86	57	0	1	0.8	0		1		1	0	0	0	361	0	0.1
87	41	0	0	-5.3	0		1		1	1	0		444	1	1.2
88	76	1	0	-0.4	0		1	1	1	1	1		286	1	0.9
89	41	0	1	-0.4	0		1		1	1	1	0	585	0	1.2
90	62	1	1	2.3	0		1	1	1	1	0		386	1	1
91	42	0	1	0.5	0		1		1	1	0		442	1	1.2

92	63	0	1	-3.8	0		1		1	1	2	0	382	1	0.5
93	64	1	1	1.0	0		1	1	1	1	0	0	393	1	0.5
94	34	0	1	-1.0	0		1		1	1	0		399	0	1.2
95	68	0	0	2.5	0		1	1	1	1	0	0	299	1	1.0
96	43	0	0	-2.3	0		1		1	1	0		385	1	0.7
97	38	0	0	0.8	0	1	1	0	1	0	0		493	1	1.2
98	40	1	1	-0.5	0		1		1	1	1	0	392	0	1.2
99	58	0	1	1.4	0		1	1	1	1	1	0	286	0	0.7
100	65	1	0	-0.5	0		1		1	1	0		342	0	0.5
101	67	0	0	0.5	0	1	1		1	1	0	0	361	1	0.5
102	49	1	0	-1.5	0		1		1	1	1		307	1	0.6
103	42	0	1	-0.5	0		1		1	1	1	0	506	0	0.04
104	49	0	1	0.4	0		1	1	1	1	0		321	1	1
105	42	0	0	0.5	0		1		1	1	1	0	528	0	1
106	49	1	0	0.3	0		1	1	1	1	1		464	1	1
107	42	0	0	-0.3	0		1		1	1	0	0	674	0	0.1
108	42	0	0	-0.8	0		1	1	1	1	1	0	414	1	0.15
109	66	0	0	-2.3	1	1	1	1	1	1	1	0	357	1	0.6
110	57	0	0	-3.0	0		1		1	1	1	0	414	0	0.7
111	55	0	1	-3.0	0		1		1	1	0		385	1	1.2
112	37	0	1	-1.0	0		1	1	1	1	0	0	393	1	1.2
113	39	1	1	3.0	0		1		1	1	1	0	459	1	1.2
114	47	0	1	-0.5	0		1	1	1	1	0		506	1	0.7
115	54	0	0	0.0	0		1	1	1	1	1	0	443	1	0.9
116	52	0	1	-1.5	0		1	1	1	1	0		357	0	0.6
117	56	1	0	0.8	0		1	0	1	1	1	0	314	1	0.4
118	70	1	1	0.0	0		1	1	1	0	0	0	305	1	0.9
119	67	1	0	1.5	0	1	1		1	1	0	0	287	0	0.3
120	47	1	0	0.5	0		1	1	1	1	0	0	228	0	1.2
121	61	1	1	-4.5	0		1	1	1	0	1	0	306	1	0.7
122	82	0	0	1.0	0	1	1	0	1	2	1	0	300	1	1.0
123	47	0	1	-0.5	0		1	1	1	0	1	0	328	1	1.2
124	45	1	1	0.0	0		1	1	1	0	0	0	494	1	1.0

125	54	0	0	0.0	0		1	1	1	1	1	0	461	1	0.8
126	62	0	1	2.3	0		1	0	1	0	1	0	221	1	1.2
127	60	0	1	2.5	0		1	0	1	0	1	0	528	1	0.8
128	53	0	0	0.0	0	1	1	0	1	2	1	0	428	1	0.8
129	42	0	1	1.0	0		1	0	1	1	0	0	356	1	1.2
130	69	0	0	0.0	1	1	1	0	1	2	1	0	458	1	1.2
131	59	0	1	1.8	0		1	1	1	1	0	0	499	1	0.9
132	64	0	0	-2.3	0		1		1	1	0	0	328	1	0.3
133	79	0	1	0.5	0		1	0	1	1	1	0	179	1	0.7
134	45	1	0	2.2	0		1	1	1	1	0	0	499	0	1.0
135	72	0	1	-2.3	0		1	1	1	0	0	0	286	1	0.7
136	51	1	0	1.5	0		1		1	0	0	0	307	1	1.2
137	41	0	0	-4.0	0		1	1	1	1	1	0	464	1	1
138	53	0	1	-0.3	0		1	0	1	1	1	0	357	1	0.8
139	47	0	1	-1.0	0		1	1	1	1	0	0	464	1	1.2
140	60	1	0	-6.5	0		1	1	1	1	0	0	357	0	0.5
141	61	0	0	2.0	0		1	0	1	1	0	0	464	1	1.2
142	48	1	0	-0.3	0		1	1	1	0	1	0	414	1	1.2
143	43	0	1	0.0	0		1	0	1	1	0	0	371	1	0.8
144	51	0	1	-1.0	0		1	0	1	1	0	0	402	1	0.6
145	47	0	0	0.0	0		1	1	1	1	0	0	357	1	1
146	54	1	1	-3.5	0		1	1	1	1	1	0	499	0	0.7
147	76	0	1	3.3	0		1	0	1	1	1	0	349	0	0.8
148	41	0	0	-2.0	0		1	0	1	1	0	0	321	1	1.2
149	61	0	1	0.3	0		1	0	1	1	1	0	336	1	0.3
150	64	0	1	-1.0	0		1	1	1	1	0	0	321	1	0.8
151	72	0	1	0.0	0		1	0	1	1	1	0	321	1	0.7
152	57	0	0	-1.0	0		1	0	1	1	0	0	285	1	1
153	66	0	0	-1.8	0	1	1	0	1	2	1	0	335	1	1
154	45	0	0	-3.8	0	1	1	0	1	1	0	0	357	1	1
155	48	1	0	-0.3	0		1	1	1	1	0	0	221	1	1.2
156	43	0	1	-0.3	0		1	0	1	1	1	0	478	1	0.7
157	63	1	0	-1.4	0		1			1	0		343	0	0.9

158	43	0	0	0.0	0		1			1	1		250	0	1.2
159	42	0	0	0.0	0		1			1	0	0	357	1	1
160	41	0	1	-1.0	1		1			2	1		536	0	0.15
161	49	0	0	-0.4	0		1	0	1	1	0	0	321	1	1.2
162	48	0	1	-0.8	0		1	0	1	0	1	0	286	1	1.2
163	65	0	1	3.9	0		1	1	1	0	1	0	279	1	0.9
164	56	1	1	-6.3	0		1	1	1	1	1	0	321	1	0.7
165	53	0	0	-1.5	0		1	0	1	0	1	0	286	1	0.7
166	63	1	0	-0.8	0		1	1	1	0	1	0	257	1	0.8
167	54	0	1	-3.3	0		1	0	1	0	0	0	328	1	0.2
168	49	0	0	-0.3	0		1	0	1	0	1	0	521	1	0.5
169	47	0	0	-0.6	0		1	1	1	0	0	0	343	1	1.2
170	45	0	0	0.1	0		1	1	1	1	0		423	1	1
171	56	0	1	-2.5	0		1	0	1	1	0		217	1	0.2
172	62	0	0	2.4	0		1	1	1	0	0		457	1	1
173	57	1	0	0.6	0		1	1	1	0	0		350	0	0.6
174	65	1	0	3.0	0		1	1	1	0	0		284	1	0.6
175	56	0	0	-0.1	1		0	1	1	2	1		578	1	0.3
176	67	1	0	-0.3	0		1	1	1	0	0		239	1	1
177	63	0	0	0.3	0		1	1	1	0	0		258	1	0.3
178	61	0	0	1.5	0		1	1	1	0	1		372	1	1
179	55	1	0	-1.3	0		1	1	1	1	0		364	1	0.4
180	85	0	0	0.8	1	1	0	1	1	0	2		601	1	0.08
181	52	0	0	-0.4	0	1	1	1	1	2	1		547	1	0.6
182	67	0	0	2.3	1	1	0	0	1	2	2		405	1	1
183	61	0	0	-0.4	1	1	0	1	1	0	2		406	1	1
184	49	0	1	-2.0	0		1	1	1	2	2		630	1	0.5
185	56	0	0	-0.1	0		1	0	1	0	0		460	1	0.7
186	59	0	0	-0.5	0		1	1	1	2	1		449	1	0.9
187	58	1	0	-1.8	0		1	1	1	0	2		325	1	0.5
188	76	0	0	0.4	0		1	1	1	2	2		550	1	1.2
189	54	1	0	-0.1	1	1	0	1	1	0	2		516	0	0.4
190	71	0	1	1.9	0		1	1	1	0	0		243	1	0.2

191	52	0	0	-3.4	0		1	0	1	2	1		381	1	1
192	50	0	0	-1.8	0		1	1	1	0	0		369	1	0.9
193	61	0	1	0.1	0		1	1	1	0	0		527	1	0.2
194	65	0	0	-3.4	0		1	1	1	0	0		432	1	0.9
195	57	1	0	0.6	0		1	1	1	0	0		357	0	0.9
196	61	0	0	1.5	0		1	1	1	0	1		372	1	1
197	69	0	0	2.3	0		1	1	1	0	1		413	0	0.5
198	49	0	0	-2.6	0		1	1	1	1	0		213	1	0.7
199	47	0	0	-4.1	0		1	1	1	0	0		421	1	0.8
200	60	0	1	-0.5	0		1	1	1	0	0		333	1	0.7
201	40	0	0	-1.5	0	1	1	1	1	0	0		547	1	1.2
202	48	0	1	-2.6	0		1	1	1	0	1		449	1	0.7
203	62	1	0	1.5	0	1	1	1	1	0	1		256	1	0.7
204	63	0	0	2.0	0		1	0	1	0	0		319	1	0.6
205	66	0	0	2.0	0	1	1	1	1	0	1		401	1	0.7
206	68	0	0	1.6	1	1	0	1	1	0	1		475	1	1
207	47	0	1	-3.5	0		1	1	1	1	0		422	1	0.5
208	77	0	1	-1.0	0		1	1	1	1	1		352	1	0.3
209	70	0	1	0.0	0		1	1	1	0	0		560	1	0.7
210	44	0	0	1.1	0		1	1	1	1	0		482	1	0.2
211	79	0	0	-0.9	0		1	1	1	1	0		259	1	0.8
212	82	1	0	-0.8	0		1	1	1	0	0		325	1	0.6
213	48	0	1	-7.4	0		1	1	1	0	0		409	1	1
214	62	1	0	1.3	0		1	1	1	0	1		410	1	1.2
215	56	1	0	-1.3	0		1	1	1	1	0		364	1	0.4
216	64	1	1	2.3	1		1			0	1			0	0.5
217	51	0	1	1.0	1		1	0		0	1			1	0.5
218	31	0	1	0.3	0		1	1	1	1	0			0	0.8
219	36	0	1	-0.9	0		1	1		0	0			0	0.8
220	49	0	0	-2.3	0		1	1	1	1	0			1	0.6
221	74	1	1	-1.0	0		1			2	1			0	0.3
222	41	1	1	0.5	0		1	1	1	1	0			1	0.6
223	69	0	0	3.0	0		1	1	1	1	1			1	0.7

224	49	0	0	0.5	0		1		1	2	1			1	0.8
225	39	0	1	-0.5	0		1		1	1	0			1	1.2
226	40	1	0	-1.5	0	1	0		1	2	1			0	1.2
227	37	0	1	-3.3	0		1		1	0	0			0	1
228	63	0	1	1.4	0		1	0	1	0	0			1	1.2
229	60	0	0	0.3	0		1		1	2	1			1	0.8
230	52	1	1	-3.6	0		1		1	1	0		216	0	1
231	48	0	1	0.3	0		1	1	1	1	0			0	0.7
232	38	0	0	0.2	0	1	0		1	1	0			0	1.2
233	72	1	1	2.0	0		0		1	0	0			1	0.5
234	57	0	1	-1.5	0		1		1	1	0			1	0.7
235	50	0	1	-1.4	0		1		1	1	0			1	1
236	39	0	1	-1.3	0		1	0	1	1	0			1	1
237	68	1	1	-1.4	0		1		1					1	1.2
238	40	0	1	0.5	0		1		1	1	0			1	0.9
239	43	1	0	0.0	0	1	0		1					1	1
240	66	0	0	-1.4	0		1		1	0	0			1	0.6
241	52	0	0	0.0	0		0	1	1	1	1			1	1.2
242	77	0	1	0.0	1		1	NA	1	0	1	0	420	1	1
243	69	1	1	0.8	1		1	1	1	0	1	1	403	1	1.5
244	66	0	1	-7.0	0		1	0	1	0	0	0	403	1	1.2
245	62	0	0	-4.1	0		1	1	1	0	0	0	237	1	1.5
246	59	0	0	-3.3	0		1	0	1	0	1	1	185	1	1.2
247	57	0	0	0.5	0		0	1	1	0	1	1	402	0	1.5
248	54	0	1	-5.3	0		1	0	1	0	1	0	202	1	0.9
249	54	0	1	-1.1	0		1	0	1	0	0	0	312	1	0.6
250	52	0	0	-0.6	0		1	0	1	0	2	0	561	0	0.7
251	51	0	0	-2.4	0		1	1	1	0	0	0	243	1	0.7
252	51	0	0	-1.1	0		1	0	1	2	1	0	357	1	1.2
253	51	1	1	-2.5	0		1	1		2	1	0	316	1	0.2
254	49	0	0	0.3	0		1	0	1	1	0	0	253	1	1.2
255	47	0	1	-1.5	0		1	0	1	0	0	1	519	0	1.2
256	47	0	1	2.3	0		1	0	1	0	0	0	315	1	1.2

257	46	0	0	-1.3	0		1	0	1	2	1	0	364	1	0.5
258	47	0	1	0.3	0		1	0	1	0	0	1	278	1	1.5
259	45	0	1	-4.8	0		1	0	1	0	0	1	239	1	1.2
260	45	0	1	-0.8	0		1	0	1	0	0	1	495	1	1.5
261	44	0	1	-0.5	0	1	1	0	1	0	1	0	541	1	1.2
262	43	0	0	0.5	0		1	1	1	0	0	1	355	0	1.5
263	43	0	0	-5.3	0	1	1	1	1	0	1	1	500	1	1.2
264	40	0	0	-0.8	0	1	1	0	1	0	1	0	495	0	1.2
265	40	0	1	-0.8	0		1	0	1	2	1	0	498	0	0.9
266	40	0	0	0.8	0		1	1	1	0	1	1	248	0	0.8
267	40	0	0	-4.3	0		1	1	0	1	0	0	284	0	1
268	39	0	0	-4.0	0		1	0	1	0	1	1	357	0	0.7
269	39	0	0	-3.6	0		1	1	1	0	0	0	173	1	1
270	39	0	0	-6.8	0		1	0	1	0	0	1	268	0	1.5
271	38	0	0	0.3	0	1	0	0	1	0	0	0	434	0	1.5
272	35	0	1	-0.4	0		1	0	1	1	0	1	466	0	0.6
273	33	1	0	0.3	0		1	1	1	1	0	1	315	0	1.2
274	43	0	1	-0.8	0		0	1	0	0	2	0	449	0	0.7
275	39	0	1	-0.5	0		1	0	1	0	0	1	429	1	1
276	54	0	0	-0.8	0		1	0	0	0	2	1	269	0	0.3
277	40	0	1	0.5	0		1	0	1	0	0	1	459	1	1.5
278	46	0	1	0.5	0		1	1	1	1	1	1	602	1	1.2
279	34	1	1	0.5	0		1	1	1	1	0	1	578	1	1.2
280	44	1	1	0.1	0		1	1	1	0	0	1	368	1	1.2
281	72	0	0	3.3	0		1	1	1	0	2	0	288	1	1
282	51	0	0	-1.0	0		1	0	1	0	0	0	366	1	0.4
283	71	1	0	0.9	0		0	1	1	0	0	1	398	1	1
284	42	0	0	0.3	0		1	0	1	0	0	1	341	1	1.2
285	54	0	0	-3.8	0		1	1	1	1	0	1	279	1	1
286	52	1	1	-2.8	0		1	1	1	1	0	0	294	1	1
287	51	0	0	-2.0	0		1	0	1	0	2	0	389	1	0.6
288	48	0	0	1.0	0		1	0	1	0	2	0	488	1	1.2
289	51	1	1	0.4	0		1	1	1	0	1	1	489	1	1.2

290	55	0	0	0.1	0		1	0	1	1	0	0	414	1	1
291	47	0	1	-1.0	0		1	1	1	1	0	1	289	1	1
292	40	0	0	0.0	0		1	0	1	0	0	1	375	1	1.2
293	64	0	1	-1.8	0		1	0	1	0	0	1	296	1	1.2
294	70	0	0	4.8	0		1	1	1	0	0	0	414	1	1.2
295	35	0	0	0.0	0	1	1	1	1	0	1	1	414	1	1.5
296	43	1	0	-8.0	0		1	1	1	0	2	1	232	1	1.2
297	46	0	1	1.0	0		1	0	1	0	1	0	407	1	1.2
298	49	0	1	0.8	0		1	0	1	0	0	1	466	1	1.2
299	59	1	1	0.3	0	1	0	0	1	0	1	0	261	1	0.9
300	50	0	1	0.5	0		1	0	1	0	0	1	235	1	1.2
301	63	0	0	-2.0	0		1	0	1	2	2	0	300	1	0.4
302	75	0	0	2.0	0	1	1	0	1	0	2	0	315	1	0.4
303	49	0	0	-0.6	0		1	1	1	2	2	0	629	1	1
304	63	0	0	-2.5	0		1	1	1	0	1	0	345	1	1.5
305	43	0	1	0.1	0		1	0	1	0	1	1	375	0	0.7
306	42	0	0	-5.0	0		1	0	1	0	0	1	347	1	1
307	65	1	0	-0.5	0		1	1	1	0	2	0	500	1	0.5
308	66	1	1	1.0	0		1	1	1	2	2	1	275	0	0.8
309	55	0	1	-1.5	0		1	0	0	0	0	1	403	1	1
310	43	0	1	0.5	0		1	0	1	0	0	1	339	1	1.5
311	48	1	1	-6.0	0		1	1	1	0	1	0	348	1	0.7
312	51	0	0	-0.4	0	1	1	1	1	2	1	1	301	0	1
313	49	0	0	0.9	0		1	0	1	2	2	0	497	1	0.3
314	60	0	1	-0.5	0		1	0	1	0	2	1	336	0	0.2
315	61	0	1	-1.1	0		1	0	1	0	1	0	325	1	0.8
316	48	0	1	-3.5	0		1	0	1	0	0	0	257	0	1.5
317	55	0	1	-3.4	0		1	1	1	0	2	1	347	1	1.2
318	47	0	0	-7.3	0		1	0	1	2	2	1	222	1	0.6
319	52	0	1	0.0	0		1	1	1	0	2	0	637	1	1.2
320	67	0	1	1.9	0		1	0	1	0	1	0	373	1	1.2
321	41	1	0	-0.3	1	1	0	1	1	2	2	0	555	0	0.4
322	64	0	1	5.5	0		1	1	1	0	0	1	246	1	0.9

323	63	1	1	-0.5	0		1	1	1	2	2	0	346	1	0.8
324	44	1	1	-3.5	0		1	1	1	1	1	1	246	1	0.8
325	48	0	1	-6.0	0		1	1	1	0	1	0	448	1	1.2
326	42	0	0	0.5	0		1	0	1	0	0	1	460	1	0.4
327	38	0	1	-1.5	0		1	0	1	1	0	1	268	0	1
328	63	0	0	1.3	0		1	0	1	2	1	1	402	1	0.6
329	54	0	1	-2.3	1		1	0		0	2	0	583	1	1
330	55	0	0	-2.0	0		1	0	1	2	2	0	492	1	1.2
331	45	0	0	-3.0	0		1	1		0	0	0	438	1	0.9
332	44	0	0	-1.6	0		1	0	1	1	0	0	378	1	0..5
333	41	0	0	0.6	0		1	0	1	0	0	0	591	1	1.5
334	53	0	1	0.5	0		1	1	1	0	0	0	305	1	1.5
335	35	0	0	-0.3	0		1	0	1	0	0	1	340	1	0.2
336	52	0	0	-0.5	0		1	1	1	0	1	0	409	1	0.9
337	53	0	1	2.0	0		1	0	1	2	1	1	451	0	1
338	51	0	0	-1.4	0		1	0	1	1	0	1	379	1	0.6
339	43	0	1	0.5	0		1	1	1	0	0	1	437	1	0.9
340	46	0	0	-0.1	0		1	0	1	1	0	1	437	1	0.1
341	45	0	0	0.0	0		1	1	1	0	0	0	437	1	1.5
342	41	1	0	0.0	0	1	0	0	1	0	0	1	552	1	1.2
343	56	0	0	-3.0	0		1	0	1	0	1	1	287	1	0.9
344	29	0	0	-1.8	0		1	1	1	0	1	0	577	1	0.9
345	61	0	1	0.5	0		1	0	1	0	2	0	389	1	0.9
346	57	0	1	-0.9	0		1	0	1	0	2	0	389	1	1.2
347	57	0	0	-0.5	0		1	0	1	0	0	0	366	0	0.7
348	64	0	1	-5.1	0		1	1	1	0	0	1	323	1	0.5
349	56	0	0	1.3	0		1	1	1	1	0	0	327	0	0.6
350	60	0	1	-1.5	0		1	1	1	0	0	1	504	1	1
351	64	0	0	1.9	0		1	1	1	0	2	0	298	1	1.5
352	45	1	1	-4.9	0		1	1	1	1	1	1	216	1	1.2
353	67	0	0	0.3	0		1	1	1	2	1	1	370	1	0.2
354	72	0	0	1.3	0		1	0	1	0	0	0	370	1	1.2
355	46	0	1	0.3	0		0	1	1	0	1	0	438	0	1.5

356	37	0	1	1.0	0		1	0	1	2	2	0	573	1	0.6
357	48	0	0	-7.3	0		1	不明	1	2	1	0	398	0	1
358	60	0	0	-0.1	0		1	1	1	1	0	1	334	1	0.9
359	53	1	1	-5.4	0		1	1	1	0	0	1	446	1	1.2
360	47	0	0	-4.5	0		1	0	0	0	1	1	446	1	1.2
361	57	0	1	3.0	0		1	1	1	0	0	1	511	1	0.9
362	68	1	0	0.6	0		1	1	1	0	0	0	208	0	1.2
363	72	0	1	0.0	0		0	1	1	0	1	0	555	1	0.9
364	48	1	1	-1.0	0		1	0	1	2	2	0	445	1	0.4
365	83	1	0	0.5	1		1	1	1	0	2	1	417	0	0.7
366	56	1	0	1.8	1	1	1	0	1	0	2	0	391	0	0.3
367	51	1	0	-1.3	1	1	0	0	1	0	2	0	520	1	1
368	71	1	1	0.5	0		1	1	1	0	0	1	455	0	1.2
369	40	0	1	-0.1	0		1	0	1	0	0	1	432	1	1
370	58	1	1	-0.1	0		0		1	1	0			1	0.7
371	61	1	1	-0.1	0		1		1	0	1			1	0.6
372	40	0	1	-2.8	0		1		1	0	0			1	1.0
373	56	1	0	-0.5	0		1		1	0	0			1	0.2
374	52	0	1	-2.4	0		1		1	0	0			1	0.5
375	44	0	0	-0.6	0		1		1	0	0			1	1.2
376	66	1	0	1.9	0		1		1	1	0			0	0.7
377	64	0	0	1.4	0		1		1	0	0			1	0.5
378	50	1	0	-0.4	0		1		1	0	0			1	0.8
379	48	0	1	1.1	0		1		1	0	1			0	0.3
380	66	0	1	3.6	0		0		1	0	1			1	0.1
381	64	1	0	0.3	1		1			0	2			1	0.8
382	29	0	1	-6.0	0		1			1	0			1	0.5
383	76	0	1	0.4	0		1			0	2			1	0.4
384	59	0	0	-0.8	0		1	1	1	1	1	0	253	1	1
385	61	0	1	-3.5	0		1	1	1	0	0	1	354	1	0.6
386	41	0	1	0.5	0		1	0	1	1	1	0	177	0	0.5
387	64	0	0	1.5	0		1	0	1	0	0	0	557	1	0.5
388	61	0	0	-2.5	0		1	1	1	1	1	0	253	1	0.9

389	77	0	0	3.0	0		1	1	1	0	0	1	633	1	0.6
390	72	0	1	3.0	0		1	1	1	0	0	0	253	1	0.6
391	47	1	1	-1.0	0		1	1	1	0	0	1	326	1	0.9
392	46	1	0	0.0	0		1	1	1	0	2	0	203	1	1.2
393	68	0	0	4.3	0		1	0	1	1	2	0	658	0	0.4
394	46	0	0	0.0	0		1	0	1	2	1	0	456	0	1.2
395	32	0	1	-0.5	0		1	0	1	0	0	0	506	1	0.7
396	53	1	0	0.3	0		1	1	1	1	0	0	557	1	0.6
397	78	1	0	4.0	0		1	1	1	0	0	0	608	1	0.2
398	40	0	0	-1.0	0		1	0	1	0	0	0	405	1	0.4
399	65	0	0	1.8	0		1	0	1	0	0	0	253	1	0.7
400	51	0	0	-1.3	0		1	1	1				759	1	0.5
401	49	1	1	-2.5	0		1	1	1	1	1	0	734	1	0.8
402	57	0	1	0.8	1		1	1	1	2	1	0	835	1	0.5
403	38	0	1	-0.5	0		1	0	1				177	1	0.7
404	55	0	0	1.3	0		1	0	1	0	0	0	177	1	0.7
405	48	0	1	0.3	0		1	1	1	1	0	0	709	1	0.6
406	67	0	1	3.0	0		1	1	1	1	0	1	152	1	0.8
407	76	1	1	-0.5	0		0	1	1	1	0	0	401	1	0.8
408	35	0	0	-0.3	0		1	0	1	0	0	1	400	1	0.4
409	54	1	0	-0.5	0		1	1	1	1	0	0	314	1	1
410	42	0	0	-0.8	0		1	1	1				582	1	0.7
411	53	0	0	1.3	0		1	0	1	0	0	0	228	1	0.8
412	41	0	0	0.3	0		1	1	1	1	0		532	0	2
413	41	0	1	5.8	0		1	0	1	1	1	0	608	1	0.6
414	75	1	1	-0.5	0		1	1	1	1	0	0	734	1	0.3
415	48	1	1	-0.3	0		1	1	1				253	1	0.5
416	54	1	1	-1.0	0		1	0	1				506	1	0.8
417	45	1	1	-0.3	0		1	0	1	1	1	0	228	1	0.4
418	40	0	1	-2.3	0		1	0	1	1	1	0	127	1	0.2
419	52	1	0	0.5	0		0	1	1	1	0	0	152	1	1.2
420	52	0	1	-0.3	0		1	0	1	0	0	0	506	1	0.6
421	66	0	1	-2.8	0		1	0	1	1	2	0	253	1	1

422	50	1	0	2.8	1		1	0	1	1	2	0	177	1	0.7
423	56	0	1	-3.0	0		0	1	0				228	1	0.15
424	53	0	1	1.0	0		1	1	1	1	0	0	203	1	0.6
425	59	0	1	1.5	0		1	1	1	1	0	0	608	1	0.8
426	70	1	0	2.0	0		1	1	1	1	1	0	304	0	0.7
427	73	1	1	0.8	0		1	1	1	0	0	0	303	0	0.9
428	48	1	0	+8.25	0		1	1	1	2	2		372	1	0.8
429	81	0	1	−0.50	0		1		1	0	2		323	0	0.8
430	35	0	0	−1.75	0		0	1	1	0	1		385	0	1.5
431	53	0	1	0.0	0		1	0	1	0	2		314	0	0.3
432	46	0	0	−6.50	0		1	1	1	2	2		397	1	0.7
433	53	0	0	−2.75	0		0	0	1	0	0		399	1	1.2
434	72	1	0	+1.00	0		1	1	1	0	2		416	1	0.6
435	68	0	0	+1.75	0		1	0	1	0	1		379	1	1.2
436	69	1	1	+2.00	0		1	1	1				460	0	1.0
437	42	0	1	−0.50	0		1	0	1	0	0		357	0	1.2
438	53	0	0	+1.75	0	1	1	0	1	0	1		356	0	0.7
439	66	0	0	−1.25	0		1	0	1	0	0		348	1	0.9
440	48	0	0	−0.25	0		1	1	1	0	0		454	1	1.5
441	60	0	1	−0.25	0		1	0	1	0	1		458	0	0.9
442	49	0	0	−0.75	0		1	0	1	0	1		458	1	1.2
443	50	0	0	+1.00	0		1	0	1	0	0		378	1	1.2
444	35	0	0	−1.00	0		1	0	1	0	1		456	0	1.5
445	56	0	1	−2.25	0		1	0	1	0	0		414	1	0.7
446	38	0	1	−0.50	0		1	0	1	2	1		395	1	1.0
447	48	0	0	−2.75	0		1		1	0	1		247	1	1.0
448	47	0	0	−0.25	0		1	1	1	0	0		356	1	0.9
449	42	0	1	−5.50	0		1	0	1				310	1	0.9
450	43	1	0	+1.75	0	1	1	1	1	0	0		476	0	1.2
451	63	0	0	-0.4	0		1		1	0	1	0	343	1	0.7
452	89	0	0	-0.3	0	1	1		1	0	2	0	544	1	0.3
453	52	1	0	2.5	0	1	1		1	0	0	0	289	1	0.8
454	40	0	1	-0.6	0		1		1	1	0	0		1	0.8

455	39	1	0	-0.3	0		1		1	1	0	0		0	1.5
456	70	0	0	1.8	0		1		1	0	1	0		1	1
457	70	0	1	1.5	0		1		1	0	0	0		1	1
458	44	1	0	-1.8	0		1		1	1	0	0		1	0.8
459	52	0	0	-0.5	1	1	0		1	0	2	0		1	1.5
460	49	1	0	0.3	0		1		1	0	2	0		1	1
461	56	1	0	1.0	0		1		1	0	2	0		1	0.5
462	54	0	0	-1.0	0	1	1		1	0	0	0		0	1
463	55	0	0	-1.0	0		1		1	0	2	0	215	0	0.5
464	49	0	1	-5.8	0		1		1	1	0	0	327	1	0.9
465	54	1	0	-1.0	0		1		1	0	0	0		1	0.9
466	46	0	1	0.4	0		1		1	0	0	0		1	0.4
467	53	1	1	2.5	0		1		1	0	0	0		1	1.2
468	55	0	1	-6.5	0		1		1	0	1	0	250	1	0.9
469	52	1	0	-4.5	0		1		1	0	0		306	1	1
470	42	0	1	-4.8	0		1		1	0	0	0	555	1	0.8
471	60	1	0	-0.8	0		0			0	0	0	398	1	0.7
472	38	0	1	2.3	1		0		1	0	1	0	640	1	0.7
473	57	0	0	0.4	0		0		0	0	0	0	333	1	0.9
474	59	1	1	1.0	0		0		1	0	1	0	295	1	0.6
475	55	1	0	0.8	0		0		1	0	0	1	346	1	0.9
476	44	0	0	0.5	0		0		1	0	0	0	412	1	1
477	54	0	0	1.3	0		0		0	0	0	0	468	1	1.2
